# DNA Barcoding Using 18S rRNA Gene Fragments for Identification of Tick-Borne Protists in Ticks in the Republic of Korea

**DOI:** 10.3390/pathogens13110941

**Published:** 2024-10-29

**Authors:** Badriah Alkathiri, Subin Lee, KyuSung Ahn, So Youn Youn, Mi-Sun Yoo, Hyang-Sim Lee, Yun Sang Cho, Jaeyun Jung, Kwangwon Seo, Soochong Kim, Rika Umemiya-Shirafuji, Xuenan Xuan, Dongmi Kwak, SungShik Shin, Seung-Hun Lee

**Affiliations:** 1 College of Veterinary Medicine, Chungbuk National University, Chungbuk 28644, Republic of Korea; badara25.ba@gmail.com (B.A.); tnqls7488@naver.com (S.L.); vetskw16@cbnu.ac.kr (K.S.); skim0026@cbnu.ac.kr (S.K.); 2 BIOREEDS Research Institute, College of Veterinary Medicine, Chonnam National University, Gwangju 61186, Republic of Korea; bonoboy1@naver.com (K.A.); sungshik@jnu.ac.kr (S.S.); 3 Laboratory of Parasitic and Honeybee Diseases, Bacterial Disease Division, Department of Animal & Plant Health Research, Animal and Plant Quarantine Agency, Gimcheon 39660, Republic of Korea; syyoun@korea.kr (S.Y.Y.); msyoo99@korea.kr (M.-S.Y.); leehs76@korea.kr (H.-S.L.); choys@korea.kr (Y.S.C.); 4 Department of Biochemistry, College of Medicine, Chungbuk National University, Chungbuk 28644, Republic of Korea; jaeyunj@chungbuk.ac.kr; 5 National Research Center for Protozoan Diseases, Obihiro University of Agriculture and Veterinary Medicine, Obihiro, Hokkaido 080-8555, Japan; umemiya@obihiro.ac.jp (R.U.-S.); gen@obihiro.ac.jp (X.X.); 6 College of Veterinary Medicine, Kyungpook National University, Daegu 41566, Republic of Korea; dmkwak@knu.ac.kr

**Keywords:** next-generation sequencing, tick-borne pathogen, protozoa, tick, vector, metabarcoding

## Abstract

The objective of this study was to evaluate the diversity and prevalence of tick-borne protists in the Republic of Korea via DNA barcoding using 18S rRNA gene fragments and PCR. Between 2021 and 2022, questing ticks were collected using the flagging method, with a total of 13,375 ticks collected and pooled into 1003 samples. Of these, 50 tick pools were selected for DNA barcoding targeting the V4 and V9 regions of 18S rRNA using the MiSeq platform. A taxonomic analysis of the amplicon sequence variants identified three genera of protozoa, namely *Hepatozoon canis*, *Theileria luwenshuni*, and *Gregarine* sp. However, the number and abundance of protists detected were different depending on the primer sets, and *T. gondii* was not identified in DNA barcoding. Furthermore, conventional PCR confirmed the presence of *H. canis*, *Toxoplasma gondii*, *T. luwenshuni*, and *Theileria* sp. in the collected ticks. This study identified *H. canis* and *T. gondii* in *Ixodes nipponensis* for the first time. It demonstrated that the results of DNA barcoding using 18S rRNA gene fragments can vary depending on the primer sets and further optimization is required for library construction to identify tick-borne protists in ticks. Despite these limitations, the findings highlight the potential of DNA barcoding using 18S rRNA gene fragments for screening the diversity of tick-borne protists in ticks.

## 1. Introduction

Ticks are blood-sucking ectoparasites that act as vectors for a variety of pathogens, including bacteria, viruses, and parasites. Previous studies have demonstrated that ticks are carriers of pathogenic bacteria, including *Anaplasma*, *Rickettsia*, *Ehrlichia*, and *Borrelia*, as well as endosymbiotic bacteria such as *Coxiella*-like endosymbionts and *Wolbachia*. Additionally, they have been identified as carriers of viruses, such as the severe fever with thrombocytopenia syndrome virus (SFTSv) and tick-borne encephalitis virus [1,2]. It is also noteworthy that ticks carry protozoan parasites belonging to the order Piroplasmida, specifically the genera *Babesia* and *Theileria*. In addition to acting as a vector for tick-borne pathogens, ticks also serve as a final host for *Hepatozoon* spp., playing a crucial role in the transmission of these parasites [3,4]. In addition to the aforementioned tick-borne pathogens, recent studies have suggested that ticks may act as a reservoir host for *Toxoplasma gondii* [5,6]. Furthermore, certain nematodes, such as the genus *Cercopithifilaria*, are known to be transmitted by ticks [7].

Microscopic examination has historically been the primary diagnostic method for identifying tick-borne pathogens. However, due to the low sensitivity of microscopic examination and the difficulties associated with species identification, the diagnosis of tick-borne pathogens using microscopy has encountered significant challenges [8]. As an alternative to microscopic examination, molecular methods such as polymerase chain reaction (PCR) have proved invaluable for the identification of tick-borne pathogens, providing molecular characteristics according to inter- or intra-species differences among tick-borne pathogens [4]. While PCR is a powerful tool for the detection of tick-borne pathogens, it is labor-intensive work that requires the use of known primer sets for pathogen detection and presents challenges in dealing with multiple genes or samples [9].

The advent of next-generation sequencing (NGS) has facilitated comprehensive taxonomic analyses in diverse samples, offering a detailed perspective on microbial diversity, even in microbial populations with low abundance [2]. The application of NGS in the field of bacteria has facilitated not only the analysis of bacterial communities through 16S rRNA V3–V4 region sequencing but also the analysis of metabolites [10]. Similarly, the 18S rRNA gene has been employed in numerous studies to assess the diversity of eukaryotic domains through the utilization of V1–V2, V3, V4, and V9 regions [11]. At present, the approach to DNA barcoding based on the 18S rRNA gene is not yet fully established in comparison to 16S rRNA gene analysis. Different results are shown according to the experimental conditions, including the target region, primer set, PCR conditions, and reference database [11,12,13]. Consequently, the results obtained by DNA barcoding must be validated by conventional or real-time PCR. Even though a number of studies have been conducted on the diversity of parasites based on DNA barcoding in a variety of samples, including human and animal feces [14,15], water [16], and so forth, there is a paucity of studies in this field that focus on ticks [17,18].

To date, numerous studies have identified a range of tick-borne pathogens, including *Anaplasma*, *Borrelia*, *Rickettsia*, and SFTSv, in ticks in the Republic of Korea (Korea) [2,19,20,21]. Regarding tick-borne protozoa, research has primarily focused on *Babesia* and *Theileria*, which are of significant medical and veterinary importance. However, other parasites have been largely overlooked [4,21,22]. Therefore, the objective of this study was to examine the diversity of tick-borne protists in field-collected ticks in Korea via DNA barcoding using 18S rRNA gene fragments, with the results subsequently validated through conventional PCR.

## 2. Materials and Methods

### 2.1. Tick Collection, Species Identification, Pooling, and DNA Extraction

**Figure 1:**
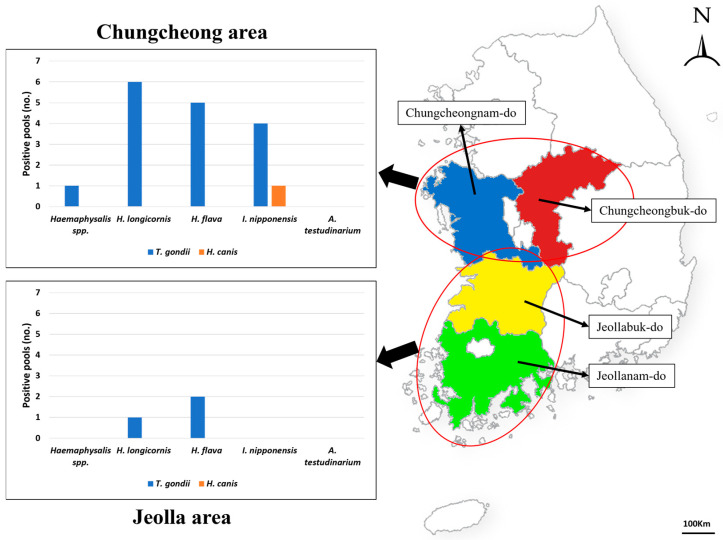
Map of the study area in Korea and the number of positive pools of *Toxoplasma gondii* and *Hepatozoon canis* by tick species. The collection sites encompass four administrative regions of Korea (Chungcheongbuk-do, Chungcheongnam-do, Jeollabuk-do, and Jeollanam-do).

Ticks were collected from March until October in 2021 and 2022 from four Korean provinces (Chungcheongbuk-do, Chungcheongnam-do, Jeollabuk-do, and Jeollanam-do) using the flagging method (Figure 1). The collected ticks were transported to the laboratory in the College of Veterinary Medicine, Chungbuk National University, Korea, and preserved in 70% ethanol at room temperature until the species and developmental stages were identified based on morphological identification features [23].

For the purpose of DNA extraction, the ticks were grouped together in the following manner: up to ten nymphs and fifty larvae. Each adult was examined based on species and sex. The pooled ticks were combined with PBS and homogenized using the bead beating method. Subsequently, the DNA was extracted using the DNeasy^®^ Blood & Tissue Kit (Qiagen, Hilden, Germany) in accordance with the manufacturer’s instructions. The concentration of DNA was determined using a spectrophotometer (DeNovix, Wilmington, DE, USA), and the samples were subsequently frozen at −20 °C for further analysis.

### 2.2. DNA Barcoding Using 18S rRNA V4 and V9 Regions

A total of 50 tick pools were selected from the 1003 tick pools for DNA barcoding using 18S rRNA gene fragments. The pools were selected according to the collected year, collected region, tick species, and developmental stage (Supplementary Table S1). To mitigate potential bias associated with varying DNA concentrations among the selected tick pools, the DNAs were normalized using Qubit™ dsDNA Quantification Assay Kits (Invitrogen, Waltham, MA, USA) and were combined as a single sample. Subsequently, the pooled DNA sample was submitted to Macrogen (Daejeon, Republic of Korea) for NGS processing.

The sequencing libraries were prepared in accordance with the Illumina 16S Metagenomic Sequencing Library protocols, with slight modifications to amplify the V4 and V9 regions of the 18S rRNA gene. In summary, the cycle conditions for the initial PCR were as follows: 3 min at 95 °C, followed by 25 cycles of 30 s at 95 °C, 30 s at 55 °C, and 30 s at 72 °C, with a final extension of 5 min at 72 °C. The universal primer pair utilized for the initial amplification of the V4 and V9 regions of 18S rRNA with Illumina adapter overhang sequences was as follows: The forward primer for the V4 amplicon PCR was 5′ TCG TCG GCA GCG TCA GAT GTG TAT AAG AGA CAG CCA GCA GCC GCG GTA ATT CC, while the reverse primer was GTC TCG TGG GCT CGG AGA TGT GTA TAA GAG ACA GAC TTT CGT TCT TGA T [24]. The forward primer for the V9 amplicon PCR was TCG TCG GCA GCG TCA GAT GTG TAT AAG AGA CAG CCC TGC CHT TTG TAC ACA C, while the reverse primer was GTC TCG TGG GCT CGG AGA TGT GTA TAA GAG ACA GCC TTC YGC AGG TTC ACC TAC [25]. Prior to library construction, the primer sets were compared in silico with 18S rRNA gene sequences from tick-borne protozoa (Supplementary Table S2).

A 10-microliter sample of the initial PCR product was employed for the final library construction, which incorporated the index through the use of the Nextera XT Indexed Primer through PCR. The second PCR cycle was conducted under the same conditions as the first, with the exception of the number of cycles, which was reduced to 10. Following each PCR process, the resulting PCR product was purified using AMPure beads (Agencourt Bioscience, Beverly, MA, USA). Subsequently, the final purified product was quantified using qPCR in accordance with the qPCR Quantification Protocol Guide (KAPA Library Quantification kits for Illumina sequencing platforms) and qualified using the TapeStation D1000 ScreenTape (Agilent Technologies, Waldbronn, Germany). Finally, the product was sequenced using the MiSeq™ platform (Illumina, San Diego, CA, USA).

### 2.3. Bioinformatics Analysis and Taxonomic Analysis

The raw sequencing data underwent processing via the removal of adapter and primer sequences, as well as trimming of forward and reverse reads to 250 and 200 base pairs, respectively, utilizing the Cutadapt v3.2 software [26]. The processes of read error correction, merging, denoising, and generating amplicon sequence variants (ASVs) were conducted using the DADA2 v1.18.0 software [27]. First, an established error model among sequence reads was used to correct errors. Then, paired-end reads were merged through overlapping, and chimera removal was performed using the consensus method of the removeBimeraDenovo function in DADA2. The resulting ASVs were employed in subsequent analyses. Each ASV was aligned to the organism exhibiting the highest degree of similarity in the corresponding reference database (NCBI_NT) using algorithms such as BLAST [28]. All statistical analyses and visualizations were conducted using R (version 4.4.0) and the RStudio environment [29].

### 2.4. Validation of DNA Barcoding Result via PCR and Phylogenetic Analysis

To corroborate the findings of the DNA barcoding analysis, three genera of tick-borne protozoa, namely *Hepatozoon* spp., *T. gondii*, and *Cytauxzoon* spp., were screened via PCR, as previously described (Table 1). Of the three genera, *H. canis* was identified in the DNA barcoding results, and the other two genera were included because their presence has been previously reported in ticks or animals in Korea [30,31,32].

The PCR was conducted using the AccuPower HotStart PCR Premix Kit (Bioneer, Daejeon, Republic of Korea) with 2 μL of template DNA, 1 μL of each forward and reverse primer (0.5 μM each), resulting in a final volume of 20 μL. Once the expected amplicon size was observed, the PCR products were sent to Macrogen (Daejeon, Republic of Korea) for bidirectional direct sequencing.

The obtained sequences were aligned using MEGA and identified to the species level through BLAST analysis based on reference sequences in the GenBank database [33]. Additionally, phylogenetic analysis was conducted to analyze the molecular characteristics of the identified tick-borne protozoa. Phylogenetic trees were constructed using MEGA (v.7.0) based on the maximum likelihood method and Tamura 3-parameter model with 500 replicates [34].

**Table 1:** List of primers utilized for the detection of tick-borne protozoa in ticks.

Pathogen	Target Gene	Primer	Sequence (5′ to 3′)	Size (bp)	Note	Reference
*Hepatozoon* spp.	18S rRNA	HepF	ATACATGAGCAAAATCTCAAC	~670		[35]
		HepR	CTTATTATTCCATGCTGCAG			
*Toxoplasma gondii*	B1	Tg_S1	TGTTCTGTCCTATCGCAACG	580	1st	[36]
		Tg_AS1	ACGGATGCAGTTCCTTTCTG			
		Tg_S2	TCTTCCCAGACGTGGATTTC	530	2nd	[36]
		Tg_AS2	CTCGACAATACGCTGCTTGA			
*Cytauxzoon* spp.	cox1	Th-For2	TGGYTKGCTTATTGGTTTGG	1966	1st	[37]
		Piro_mt_R1	ACTTTGAACACACTGCTCG			
		Th-For2	TGGYTKGCTTATTGGTTTGG	1656	2nd	[37]
		Cytaux_260R	AATTCCCATCTCGCTATCACTTTC			

## 3. Results

### 3.1. Tick Collection and Species Identification

A total of 13,375 ticks (1003 pools) were collected and categorized, belonging to three genera and five species: *Haemaphysalis* spp., *H. longicornis*, *H. flava*, *Ixodes nipponensis*, and *A. testudinarium*. The larvae stage of *Haemaphysalis* was referred to as *Haemaphysalis* spp. due to the high morphological similarities between the species, and the exact species was not determined.

### 3.2. DNA Barcoding of 18S rRNA Gene V4 and V9 Regions

After performing NGS, a total of 310,960 reads and 292,436 reads were obtained for 18S rRNA gene V4 and V9 regions, respectively. In the initial analysis, the number of ASVs was 70 and 50 for the V4 and V9 regions, respectively. However, the majority of the reads and ASVs were derived from the tick itself, and sequences belonging to the Family Ixodidae were excluded through bioinformatics (Supplementary Table S3). In conclusion, a total of 12 ASVs were included in the taxonomic analyses for the V4 region and 13 for the V9 region, with abundances of 0.20% and 0.14%, respectively, compared to the initial abundances. Subsequently, an additional analysis was conducted on the sequences belonging to the non-Family Ixodidae (Supplementary Table S4).

**Figure 2:**
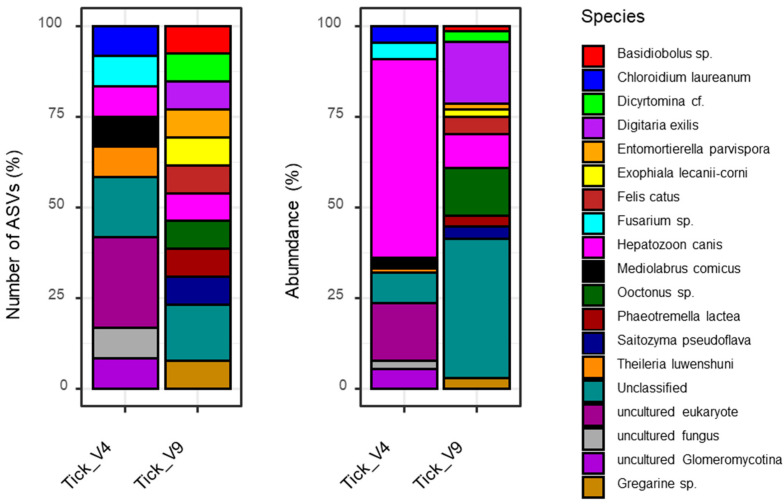
Taxonomic classification of eukaryotes in ticks based on the number of amplicon sequence variants and the relative abundance of V4 and V9 reads. Detailed information is included in supplementary Table S4.

The taxonomic analysis identified three species of protozoa: *H. canis*, *Theileria luwenshuni*, and *Gregarine* sp. (Figure 2). The presence of *H. canis* was observed in both the V4 and V9 regions, while *T. luwenshuni* and *Gregarine* sp. were identified exclusively in the V4 and V9 regions, respectively. Furthermore, a discrepancy in abundance was observed among the parasites. *H. canis* exhibited a higher abundance of 54.74% in the V4 region compared to 9.31% in the V9 region. The abundance of *T. luwenshuni* in the V4 region was 1.05%, while that of *Gregarine* sp. was 2.94% in the V9 region.

As the study targeted the 18S rRNA, it is notable that not only tick-borne protists but also other eukaryotes, such as fungi, were observed. The V4 region yielded the identification of uncultured eukaryote and uncultured fungus, while the V9 region demonstrated the presence of other eukaryotic sequences, including *Phaeotremella lactea*, *Basidiobolus* sp., and *Dicyrtomina* cf. (Figure 2).

### 3.3. Identification of Tick-Borne Protozoa by PCR

A total of one (0.10%) and 19 (1.89%) of the 1003 pools tested positive for *Hepatozoon* spp. and *T. gondii*, respectively (Table 2). However, the presence of *Cytauxzoon* was not confirmed in this study.

The prevalence of *T. gondii* varied according to the tick species. It was the most prevalent in *I. nipponensis* (6.45%, 4/62 pools), followed by *H. flava* (3.74%, 7/187 pools), *H. longicornis* (1.19%, 7/588 pools), and *Haemaphysalis* spp. (0.64%, 1/156 pools). *Hepatozoon* was identified in only one of the *I. nipponensis* pools (1.61%, 1/62).

With regard to the developmental stage, *T. gondii* demonstrated a prevalence of 4.61% (3/65 pools) in males, followed by females (2.13%, 3/141 pools), nymphs (1.9%, 12/630 pools), and larvae (0.60%, 1/167 pools). *Hepatozoon* was identified in only one male sample (1.53%, 1/65 pools).

### 3.4. Sequence Analysis and Phylogenetic Analysis

**Figure 3:**
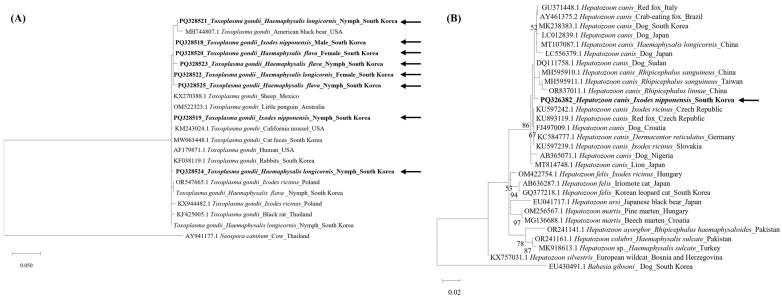
Phylogenetic analysis of (**A**) *T. gondii* based on of the B1 gene and (**B**) *Hepatozoon* spp. based on the 18S rRNA. The phylogenetic trees were constructed using the maximum likelihood method and Tamura 3-parameter model with 500 bootstrap replications. The accession numbers of GenBank with species, host, and country are described. Sequences from this study are in bold with arrows.

Fragments of the 18S rRNA gene of *Hepatozoon* spp. obtained in this study were compared with available sequences in the GenBank database via BLAST. BLAST results showed that *Hepatozoon* in this study shared 99.51% identity with previously reported *H. canis* in *I. ricinus* from Slovakia (KU597239) and the Czech Republic (KU597242), 99.31% in *H. longicornis* from China (MT107087), and 98.70% in dogs from Japan (LC556379). Furthermore, the sequence demonstrated 99.31% similarity with *H. canis* reported from dogs in Korea (MK238383 and MK238384). Phylogenetic analysis revealed that *H. canis* in this study was well-clustered with other *H. canis* and was clearly separated from other *Hepatozoon* species (Figure 3).

Of the 1003 tick pools tested, 19 tick pools were positive for *T. gondii*, of which 12 positive PCR products were successfully sequenced. Fragments of the B1 gene sequences of the analyzed samples exhibited 99.20–100% similarity to those of *T. gondii* in an American black bears from the USA (MH744807), a little penguin from Australia (OM522323), cat feces (MW063448), and rabbits (KF038119) from Korea.

## 4. Discussion

To date, NGS techniques, particularly DNA barcoding, have been employed to identify eukaryotes’ diversity, including parasites, in a range of samples. These include helminths in wild rodents [38], eukaryotes in pond water [39], protozoa in surface water [16], and parasites in wildlife [15]. Despite the disparate experimental conditions employed by various researchers, the findings consistently demonstrated the efficacy of DNA barcoding of 18S rRNA gene fragments in identifying parasite diversity across diverse sample types.

In this study, three genera of parasites, including *Hepatozoon*, *Theileria*, and *Gregarine* sp., were identified via DNA barcoding using 18S rRNA gene fragments, while two species, including *H. canis* and *T. gondii*, were identified via PCR. In addition to the current study, our previous research demonstrated the presence of *T. luwenshuni* and *Theileria* sp. in the ticks examined in this study [4]. In accordance with the results of previous studies [13,14], it can be observed that different results can be obtained according to the PCR primer sets and target region in DNA barcoding using 18S rRNA gene fragments. The results of this study demonstrated discrepancies between the DNA barcoding and PCR results.

*H. canis* is a protozoan parasite that is not transmitted via blood feeding; rather, it is transmitted by the ingestion of infected ticks which act as definitive hosts, and which feed on intermediate hosts [35]. It is thought that *H. canis* is transmitted by the brown dog tick, *Rhipicephalus sanguineus* [35]. Moreover, other tick species, including *Ixodes ricinus*, *I. canisuga*, *I. hexagonus*, *Dermacentor reticulatus*, and *Haemaphysalis concinna*, have been proposed as potential vectors of *H. canis* [40,41]. It is noteworthy that Korea is not a region endemic to *Rhipicephalus* [42]. Furthermore, none of the previous studies have identified *H. canis* in ticks collected from Korea. However, the presence of *H. canis* has been documented in the blood of dogs in Korea, which has contributed to the uncertainty surrounding the vector of *H. canis* in Korea [21,30]. In this study, the first identification of *H. canis* in *I. nipponensis* was achieved via DNA barcoding of the V4 and V9 regions of the 18S rRNA gene, with one positive case subsequently identified through PCR. Furthermore, phylogenetic analysis demonstrated that the *H. canis* identified in this study exhibited a high degree of genetic identity with the *H. canis* documented in animals and ticks in foreign countries, particularly, a close relationship with the *H. canis* isolated from dogs in Korea. In light of these findings, it can be posited that the detection of *H. canis* in ticks does not merely signify the presence of the parasite in the tick population, but rather it suggests the potential for *I. nipponensis* as a vector for *H. canis*.

*Toxoplasma gondii* is a significant apicomplexan parasite. Cats serve as definitive hosts, while most mammals can act as intermediate hosts [6]. Given that the majority of *T. gondii* infections are the result of ingestion of contaminated water, food, or raw meat, the parasite is not typically considered to be transmitted by ticks [6]. Nevertheless, recent studies indicate that ticks may play a role in the transmission of *T. gondii*, with some studies suggesting that they may serve as reservoir hosts [5,6]. Prior research in Korea has demonstrated the presence of *T. gondii* (4.1%, 13/314) in *H. longicornis* and *H. flava* [31]. Additionally, this study demonstrated the presence of *T. gondii* in various tick species, including *H. longicornis*, *H. flava*, and *I. nipponensis*. Among these tick species, *T. gondii* was first detected in *I. nipponensis*.

It is noteworthy that *T. gondii*, which was identified through PCR, was not identified through DNA barcoding using 18S rRNA gene fragments in both the V4 and V9 regions. Despite the absence of *T. gondii* identification through DNA barcoding, previous studies in Korea have demonstrated the presence of this parasite in ticks [31]. Consequently, an investigation was conducted targeting the B1 gene, which was utilized in the aforementioned study [31]. A total of 19 (1.89%) samples were identified as positive for *T. gondii* by PCR. Some of the positive samples were included in the pools used for DNA barcoding; however, the DNA of *T. gondii* was not identified in DNA barcoding. This result may be attributed to one or more of the following factors: first, the quantity of DNA included in the DNA barcoding was insufficient. Secondly, the primer sets utilized for DNA barcoding were not optimal for the detection of *T. gondii*. Indeed, Cooper et al. [43] detected *T. gondii* in the tissue of Risso’s dolphin by NGS targeting the V1–V3 hypervariable region of the 18S rRNA gene, while Moreno et al. [16] identified *T. gondii* in water by NGS targeting the V4 region of the 18S rRNA gene. Furthermore, the majority of *T. gondii* B1 gene-positive cases yielded negative results in nested PCR targeting 18S rRNA (Supplementary Table S5), indicating that the detection rate is contingent upon the PCR conditions and target gene. Although the specific experimental conditions were different from this study, PCR targeting the B1 gene was more sensitive than PCR targeting 18S rRNA [44]. It can therefore be concluded that optimization of library construction is essential for the successful application of DNA barcoding using 18S rRNA gene fragments for parasite detection.

The phylogenetic analysis of the *T. gondii* B1 gene and 18S rRNA revealed that the *T. gondii* sequences derived from ticks exhibited no significant divergence according to the host species, including cats, or geographical origin. It would be premature to draw conclusions regarding the vector competence of ticks for *T. gondii* transmission at this stage. Further studies are required to gain a better understanding of this phenomenon, particularly using experimental animals.

In a previous study, we detected *Theileria* spp. in ticks in Korea, and a subset of the ticks are also included in the current study [4]. Our previous study demonstrated a high prevalence of *Theileria*, comprising two species: *T. luwenshuni* and *Theileria* sp. Given our prior awareness of the presence of these two *Theileria* species in the tested ticks, we did not undertake a validation process via PCR on the two *Theileria* spp. However, only *T. luwenshuni* was identified, and *Theileria* sp. was not detected in the DNA barcoding results. Considering that *T. luwenshuni* was only detected in the V4 region in the DNA barcoding, and its abundance was only 3, we believe that this may be due to the low amount of *Theileria* DNA during the library construction or the low throughput of the NGS running. As Mans et al. [45] successfully identified *Theileria* spp. by amplifying the V4 hypervariable region of the 18S rRNA gene, but not using NGS, it is believed that different *Theileria* spp. can be detected by DNA barcoding after optimization of library construction.

This study employed DNA barcoding of 18S rRNA gene fragments to comprehensively analyze tick-borne protists, yet several limitations exist. A limitation of the study is that the DNA used in the barcoding was pooled, which means that ticks that were not infected with the pathogens of interest were included, resulting in a lower amount of DNA from these pathogens. In the end, this is likely to lower the potential of detection. Furthermore, the primers utilized in this study targeted the 18S rRNA gene, resulting in the majority of the reads obtained from NGS being from the tick itself rather than pathogens. In fact, 172,178 and 171,979 abundances were obtained in the V4 and V9 regions, respectively, but only 285 and 204 of those reads corresponded to non-Ixodidae. This further reduced the detectable amount of pathogens (Supplementary Tables S3 and S4). As shown in these results, DNA barcoding can detect very small amounts of ASVs, but if the amount of the pathogen’s DNA is low during library construction or low throughout NGS running, the result is that fewer pathogen-derived ASVs are generated, which limits the detection of ASVs. These limitations could be addressed by conducting DNA barcoding on individual ticks, by increasing the amount of throughput of NGS running, or by using a PCR blocker, which can prevent amplification of host DNA [46].

It is undeniable that the use of universal primer sets targeting the 18S rRNA gene resulted in the detection of non-target eukaryotes. Furthermore, primer set selection is crucial in DNA barcoding of the 18S rRNA gene because it is known that different primer sets yield different results, even in the same target region [12]. In this study, some fungal genera were identified, including *Phaeotremella* and *Basidiobolus*, which are commonly identified in natural environments and distributed worldwide [47,48]. As the objective of the study was to investigate the occurrence of tick-borne protists in ticks, these eukaryotes were excluded from the data set. However, given the stated aim of the study, using a universal primer set will prove beneficial.

## 5. Conclusions

In conclusion, the use of DNA barcoding of 18S rRNA gene fragments is an effective method for studying protozoan parasites associated with ticks. This study comprehensively identified tick-borne protists in ticks based on the DNA barcoding of the 18S rRNA gene and PCR. The results revealed the presence of *H. canis* and *T. gondii* in *I. nipponensis* for the first time. DNA barcoding is a valuable tool for identifying tick-borne protists. However, further optimization is necessary for the library construction, and the use of different primer sets should be considered to achieve comprehensive detection of tick-borne protists.

## Figures and Tables

**Table 2:** Identification of *H. canis* and *T. gondii* via PCR in ticks collected in Korea.

Tick Species	Developmental Stage	No. of Tested Tick ^1^ (Pool)	No. of Positive Pool	
			*H. canis*	*T. gondii*
*Haemaphysalis* spp. ^2^	Larva	7153 (156)	-	1 (0.64)
*H. longicornis*	Nymph	4441 (461)	-	6 (1.30)
	Male	23 (23)	-	0/23
	Female	104 (104)	-	1 (0.96)
	sub total	4568 (588)	-	7 (1.19)
*H. flava*	Nymph	1355 (153)	-	5 (4.34)
	Male	17 (17)	-	0/17
	Female	17 (17)	-	2 (11.76)
	sub total	1389 (187)	-	7 (3.74)
*I. nipponensis*	Larva	70 (6)	-	0/6
	Nymph	16 (11)	-	1 (9.09)
	Male	25 (25)	1 (4)	3 (12)
	Female	20 (20)	-	0/20
	sub total	131 (62)	1 (1.61)	4 (6.45)
*A. testudinarium*	Larva	124 (5)	-	0/5
	Nymph	10 (5)	-	0/5
	sub total	134 (10)	-	0/10
Total		13,375 (1003)	1 (0.09)	19 (1.89)

^1^ Depending on stage level, up total to 50 larvae and 10 nymphs were pooled. The adults were pooled individually. ^2^ *Haemaphysalis* spp. refers to the larva stage of *Haemaphysalis* genus due to the difficulty of morphological identification.

## Data Availability

The data presented in this study are contained within this article, and all sequences obtained in this study were submitted to the GenBank Database (Accession Nos. PQ328518-PQ328525 and PQ326380-PQ326382) or Sequence Read Archive under BioProject PRJNA1158226.

## Supplementary Materials

The following supporting information can be downloaded at: https://www.mdpi.com/article/10.3390/pathogens13110941/s1, Table S1: Information of tick pools included in this study for DNA barcoding using 18S rRNA gene fragments. Table S2: Eukaryotic universal primer sets were tested in silico with 18S rRNA gene sequences from tick-borne protozoa. Table S3: Taxonomic classification of eukaryotes in ticks according to relative abundance of V4 and V9 reads. Table S4: Taxonomic classification of eukaryotes in ticks based on the number of ASVs and abundance of V4 and V9 reads. By using bioinformatics, ASVs corresponding to Family Ixodidae were filtered out and only remaining ASVs are shown. Table S5: PCR positive samples for *T. gondii* identified by targeting B1 and 18S rRNA genes.
