# Supplementary material for: DNA Barcoding Using 18S rRNA Gene Fragments for Identification of Tick-Borne Protists in Ticks in the Republic of Korea
Source: Pathogens. 2024 Oct 29;13(11):941. doi: 10.3390/pathogens13110941 (PMC11597262; doi:10.3390/pathogens13110941)
Supplement: Supplementary file 1 [file pathogens-13-00941-s001.zip › pathogens-3243148-supplementary.pdf]

Table S1. Information of tick pools included in this study for DNA-barcoding of 18S rRNA gene.

Collection date	Region[1]	Species	Developmental stage	No. of ticks per pool	Tick ID	Results by PCR			
						Toxoplasma gondii	*Hepatozoon canis*	*Theileria luwenshuni*[2]	*Theileria* sp.[2]
20210316	CC	*I. nipponensis*	Male	1	Tick-C5		1		
20210316	CC	*I. nipponensis*	Nymph	1	Tick-C6				
20210323	CC	*H. flava*	Nymph	3	Tick-C7		1		
20210323	CC	*I. nipponensis*	Nymph	1	Tick-C17		1		
20210329	CC	*I. nipponensis*	Male	1	Tick-C21	1	1		
20210329	CC	*H. flava*	Female	1	Tick-C30				
20210330	CC	*H. flava*	Female	1	Tick-C40		1		
20210423	CC	*H. flava*	Nymph	10	Tick-C79			1	
20210518	CC	*H. longicornis*	Nymph	10	Tick-C108		1		1
20210709	CC	*I. nipponensis*	Larva	5	Tick-C202				
20210812	CC	*H. longicornis*	Female	1	Tick-C232		1	1	
20210813	CC	*H. longicornis*	Male	1	Tick-C243			1	
20210819	CC	*I. nipponensis*	Larva	6	Tick-C246				
20210923	CC	*H. flava*	Nymph	9	Tick-C272		1		
20211004	CC	*H. flava*	Male	1	Tick-C280				
20211018	CC	*I. nipponensis*	Female	1	Tick-C290				
20220311	CC	*H. flava*	Male	1	C22-1				
20220311	CC	*I. nipponensis*	Female	1	C22-2				
20220321	CC	*I. nipponensis*	Male	1	C22-16				
20220329	CC	*I. nipponensis*	Nymph	2	C22-32				
20220408	CC	*H. flava*	Male	1	C22-40				
20220408	CC	*H. longicornis*	Nymph	10	C22-51				
20220422	CC	*H. longicornis*	Nymph	10	C22-81		1		
20220422	CC	*H. flava*	Nymph	4	C22-82				
20220720	CC	*I. nipponensis*	Larva	41	C22-199				
20210315	JL	*H. flava*	Male	1	Tick-J18				
20210315	JL	*H. flava*	Nymph	10	Tick-J20		1		
20210319	JL	*H. flava*	Female	1	Tick-J27			1	
20210319	JL	*I. nipponensis*	Male	1	Tick-J28			1	
20210319	JL	*A. testidunarium*	Nymph	1	Tick-J30				
20210421	JL	*I. nipponensis*	Female	1	Tick-J65			1	
20210427	JL	*A. testidunarium*	Nymph	1	Tick-J67				

20210628	JL	*I. nipponensis*	Nymph	1	Tick-J145		
20210628	JL	*A. testidunarium*	Larva	8	Tick-J146		
20210628	JL	*A. testidunarium*	Larva	9	Tick-J152		
20210709	JL	*A. testidunarium*	Larva	37	Tick-J165		
20210729	JL	*H. longicornis*	Male	1	Tick-J180		1
20210902	JL	*H. longicornis*	Female	1	Tick-J215		
20220315	JL	*I. nipponensis*	Male	1	J22-2		
20220321	JL	*I. nipponensis*	Female	1	J22-13		
20220331	JL	*A. testidunarium*	Nymph	5	J22-34		
20220331	JL	*H. longicornis*	Nymph	10	J22-40	1	
20220416	JL	*A. testidunarium*	Nymph	2	J22-46		
20220416	JL	*A. testidunarium*	Larva	20	J22-50		
20220727	JL	*I. nipponensis*	Nymph	2	J22-158		
20220727	JL	*I. nipponensis*	Larva	12	J22-161		
20220707	JL	*H. longicornis*	Female	1	J22-165		
20220707	JL	*H. flava*	Nymph	7	J22-169	1	
20220711	JL	*H. longicornis*	Male	1	J22-174		
20220814	JL	*H. flava*	Female	1	J22-188		

[1]CC, Chungcheongbuk-do and Chungcheongnam-do; JL, Jeollabuk-do and Jeollanam-do

[2]Positivity and species of *Theileria* were confirmed in our previous study [4].

Table S2. Eukaryotic universal primer sets were tested *in silico* with 18S rRNA gene sequences from tick-borne protozoa. In this analysis, sequences from 18S rRNA of *Babesia gibsoni* (KP666162 and AB478320), *Hepatozoon canis* (MK091090 and PQ326382), *Theileria luwenshuni* (OP445729 and JX469515), *Theileria* sp. (OP445726), *Theileria orientalis* (AB520955), and *Toxoplasma gondii* (L24381 and U03070) were included.

```
              ....|....| ....|....| ....|....| ....|....| ....|....| ....|....| ....|....| ....|....| ....|....| ....|....|
                     510        520        530        540        550        560        570        580        590        600
V4F           ---------- ---------- ---------- ---------- ---------- ---------- ---------- -----CCAGC AGCCGCGGTA ATTCC-----
V4R           ---------- ---------- ---------- ---------- ---------- ---------- ---------- ---------- ---------- ----------
V9F           ---------- ---------- ---------- ---------- ---------- ---------- ---------- ---------- ---------- ----------
V9R           ---------- ---------- ---------- ---------- ---------- ---------- ---------- ---------- ---------- ----------
KP666162.1    ATT-----GT CTTGTAATCG GAATGATGGT GACGTAAAAT CTCACCAGAG TAACAATTGG AGGGCAAGTC TGGTGCCAGC AGCCGCGGTA ATTCCAGCTC
AB478320.1    ATT-----GT CTTGTAATTG GAATGATGGT GACGTAAAAT CTCACCAGAG TAACAATTGG AGGGCAAGTC TGGTGCCAGC AGCCGCGGTA ATTCCAGCTC
MK091090.1    ATTAAAATGC TTTGAAATTG CAATGATAGA AATTTAAACC CTTTTTAAAG TATCAATTGG AGGGCAAGAC TGGTGCCAGC AGCCGCGGTA ATACCAGCTC
PQ326382.1    GTTAAAATGC TTTGTAATTG GAATGATAGA AATTTAAACC CTTTTTAAAG TATCAATTGG AGGGCAAGTC TGGTGCCAGC AGCCGCGGTA ATTCCAGCTC
OP445729.1    ---------- ---------- ---------- ---------- ---------- ---------- AGGGCAAGTC TGGTGCCAGC AGCCGCGGTA ATTCCAGCTC
JX469515.1    CTT--AATGT CTTGTAATTG GAATGATGGG AATTTAAACC TCTTCCAGAG TATCAATTGG AGGGCAAGTC TGGTGCCAGC AGCCGCGGTA ATTCCAGCTC
OP445726.1    ---------- ---------- ---------- ---------- --------AG TATCAATTGG AGGGCAAGTC TGGTGCCAGC AGCCGCGGTA ATTCCAGCTC
AB520955.1    CTT--AATGT CTTGTAATTG GAATGATGGG AATTTAAACC TCTTCCAGAG TATCAATTGG AGGGCAAGTC TGGTGCCAGC AGCCGCGGTA ATTCCAGCTC
L24381.1 T    TTT--CATTT CTAGTGATTG GAATGATAGG AATCCAAACC CCTTTCAGAG TAACAATTGG AGGGCAAGTC TGGTGCCAGC AGCCGCGGTA ATTCCAGCTC
U03070.1 T    TTT--CATTT CTAGTGATTG GAATGATAGG AATCCAAACC CCTTTCAGAG TAACAATTGG AGGGCAAGTC TGGTGCCAGC AGCCGCGGTA ATTCCAGCTC
```

```
              ....|....| ....|....| ....|....| ....|....| ....|....| ....|....| ....|....| ....|....| ....|....| ....|....|
                     910        920        930        940        950        960        970        980        990       1000
V4F           ---------- ---------- ---------- ---------- ---------- ---------- ---------- ---------- ---------- ----------
V4R           ---------- ---------- ---------- ---------- ---------- ---------- ---------- ---------- ---TTAATCA AGAACGAAAG
V9F           ---------- ---------- ---------- ---------- ---------- ---------- ---------- ---------- ---------- ----------
V9R           ---------- ---------- ---------- ---------- ---------- ---------- ---------- ---------- ---------- ----------
KP666162.1    ATTCGTATTT AACTGTCAGA GGTGAAATTC TTAGATTTGT TAAAGACGAA CTACTGCGAA AGCATTTGCC AAGGACGTTT TCATTAATCA AGAACGAAAG
AB478320.1    ATTCGTATTT AACTGTCAGA GGTGAAATTC TTAGATTTGT TAAAGACGAA CTACTGCGAA AGCATTTGCC AAGGACGTTT TCATTAATCA AGAACGAAAG
MK091090.1    ATTTGTATTT AACTGTCAGA GGTGAAATTC TTAGATTTGT TAGAGACAAA CTACTGCGAA AGCATTTGCC AAAGATGTTT TCATTAATCA AGAACGAAAG
PQ326382.1    ---------- ---------- ---------- ---------- ---------- ---------- ---------- ---------- ---------- ---ACG----
OP445729.1    ATTCGTATTT AACTGTCAGA GGTGAAATTC TTAGATTTGT TAAAGACGAA CTACTGCGAA AGCATTTGCC AAGGATGTTT TCATTAATCA AGAACGAAAG
JX469515.1    ATTCGTATTT AACTGTCAGA GGTGAAATTC TTAGATTTGT TAAAGACGAA CTACTGCGAA AGCATTTGCC AAGGATGTTT TCATTAATCA AGAACGAAAG
OP445726.1    ATTCGTATTT AACTGTCAGA GGTGAAATTC TTAGATTTGT TAAAGACGAA CTACTGCGAA AGCATTTGCC AAGGATGTTT TCATTAATCA AGAACGAAAG
AB520955.1    ATTCGTATTT AACTGTCAGA GGTGAAATTC TTAGATTTGT TAAAGACGAA CTACTGCGAA AGCATTTGCC AAGGATGTTT TCATTAATCA AGAACGAAAG
L24381.1 T    ATTCGTATTT AACTGTCAGA GGTGAAATTC TTAGATTTGT TAAAGACGAA CTACTGCGAA AGCATTTGCC AAAGATGTTT TCATTAATCA AGAACGAAAG
U03070.1 T    ATTCGTATTT AACTGTCAGA GGTGAAATTC TTAGATTTGT TAAAGACGAA CTACTGCGAA AGCATTTGCC AAAGATGTTT TCATTAATCA AGAACGAAAG
```

```
              ....|....| ....|....| ....|....| ....|....| ....|....| ....|....| ....|....| ....|....| ....|....| ....|....|
                    1010       1020       1030       1040       1050       1060       1070       1080       1090       1100
V4F           ---------- ---------- ---------- ---------- ---------- ---------- ---------- ---------- ---------- ----------
V4R           T--------- ---------- ---------- ---------- ---------- ---------- ---------- ---------- ---------- ----------
V9F           ---------- ---------- ---------- ---------- ---------- ---------- ---------- ---------- ---------- ----------
V9R           ---------- ---------- ---------- ---------- ---------- ---------- ---------- ---------- ---------- ----------
KP666162.1    T--TAGGGGA TCGAAGACGA TCAGATACCG TCGTAGTCCT AACCATAAAC CATGCCGACT AGGGATTGGA GGTCGTCATT TTT--CGACT CCTTCAGCAC
AB478320.1    T--TAGGGGA TCGAAGACGA TCAGATACCG TCGTAGTCCT AACCATAAAC CATGCCGACT AGGGATTGGA GGTCGTCATT TTT--CGACT CCTTCAGCAC
MK091090.1    TTATAGGGGA TCGAAGACGA TCAGATACCG TCGTAGTCCT AACTATAAAC TATGCCGACT AGAGATTGGA GGTCGTCTTT ATAAACGACT CCTTCAGCAC
PQ326382.1    ---------- ---------- ---------- ---------- ---------- ---------- ---------- ---------- ---------- ----------
OP445729.1    T--TAGGGGA TCGAAGACGA TCAGATACCG TCGTAGTCCT AACCATAAAC TATGCCGACT AGAGATTGGA GGTCGTCAGT TTATACGACT CCTTCAGCAC
JX469515.1    T--TAGGGGA TCGAAGACGA TCAGATACCG TCGTAGTCCT AACCATAAAC TATGCCGACT AGAGATTGGA GGTCGTCAGT TTTTACGACT CCTTCAGCAC
OP445726.1    T--TAGGGGA TCGAAGACGA TCAGATACCG TCGTAGTCCT AACCATAAAC TATGCCGACT AGAGATTGGA GGTCGTCAGT TTTTACGACT CCTTCAGCAC
AB520955.1    T--TAGGGGA TCGAAGACGA TCAGATACCG TCGTAGTCCT AACCATAAAC TATGCCGACT AGAGATTGGA GGTCGTCAGT TTTTACGACT CCTTCAGCAC
L24381.1 T    T--TAGGGGC TCGAAGACGA TCAGATACCG TCGTAGTCTT AACCATAAAC TATGCCGACT AGAGATAGGA AAACGT-ATG CTT---GACT TCTCCTGCAC
U03070.1 T    T--TAGGGGC TCGAAGACGA TCAGATACCG TCGTAGTCTT AACCATAAAC TATGCCGACT AGAGATAGGA AAACGTCATG CTT---GACT TCTCCTGCAC
```

```
                 1610       1620       1630       1640       1650       1660       1670       1680       1690       1700
V4F          ---------- ---------- ---------- ---------- ---------- ---------- ---------- ---------- ---------- ----------
V4R          ---------- ---------- ---------- ---------- ---------- ---------- ---------- ---------- ---------- ----------
V9F          ---------- ---------- ---------- ---------- ---------- --CCCTGCCH TTTGTACACA C--------- ---------- ----------
V9R          ---------- ---------- ---------- ---------- ---------- ---------- ---------- ---------- ---------- ----------
KP666162.1   ATGAACGAGG AATGCCTAGT ATGCGCAAGT CATCAGCTTG TGCAGATTAC GTCCCTGCCC TTTGTACACA CCGCCCGTCG CTCCTACCGA TCGAGTGATC
AB478320.1   ATGAACGAGG AATGCCTAGT ATGCGCAAGT CATCAGCTTG TGCAGATTAC GTCCCTGCCC TTTGTACACA CCGCCCGTCG CTCCTACCGA TCGAGTGATC
MK091090.1   TTTAACGAGG AATGCCTAGT AAGCGCGAGT CATCAGCTTG CGCTGATTAC GTCCCTGCCC TTTGTACACA CCGCCCGTCG CTCCTACCGA TTGAGTGATC
PQ326382.1   ---------- ---------- ---------- ---------- ---------- ---------- ---------- ---------- ---------- ----------
OP445729.1   ---------- ---------- ---------- ---------- ---------- ---------- ---------- ---------- ---------- ----------
JX469515.1   GTGAACGAGG AATGCCTAGT ATGCGCAAGT CATCAGCTTG TGCAGATTAC GTCCCTGCCC TTTGTACACA CCGCCCGTCG CTCCTACCGA TCGAGTGATC
OP445726.1   ---------- ---------- ---------- ---------- ---------- ---------- ---------- ---------- ---------- ----------
AB520955.1   GTGAACGAGG AATGCCTAGT ATGCGCAAGT CATCAGCTTG TGCAGATTAC GTCCCTGCCC TTTGTACACA CCGCCCGTCG CTCCTACCGA TCGAGTGATC
L24381.1 T   TTCAACGAGG AATGCCTAGT AGGCGCAAGT CAGCAGCTTG CGCCGATTAC GTCCCTGCCC TTTGTACACA CCGCCCGTCG CTCCTACCGA TTGAGTGTTC
U03070.1 T   TTCAACGAGG AATGCCTAGT AGGCGCAAGT CAGCACGTTG CGCCGATTAC GTCCCTGCCC TTTGTACACA CCGCCCGTCG CTCCTACCGA TTGAGTGTTC

             ....|....| ....|....| ....|....| ....|....| ....|....| ....|....| ....|....| ....|....| ....|....| ....|....|
                 1710       1720       1730       1740       1750       1760       1770       1780       1790       1800
V4F          ---------- ---------- ---------- ---------- ---------- ---------- ---------- ---------- ---------- ----------
V4R          ---------- ---------- ---------- ---------- ---------- ---------- ---------- ---------- ---------- ----------
V9F          ---------- ---------- ---------- ---------- ---------- ---------- ---------- ---------- ---------- ----------
V9R          ---------- ---------- ---------- ---------- ---------- ---------- ---------- ---------- ---------- ----------
KP666162.1   CGGTGAATTA TTCGGACCGT GGCTTTTCCG ATTCGTCGGT TTTGCCTAGG GAAGTTTTGT GAACCTTATC ACTTAAAGGA AGGAGAAGTC GTAACAAGGT
AB478320.1   CGGTGAATTA TTCGGACCGT GGCTTTTCCG ATTCGTCGGT TTTGCCTAGG GAAGTTTTGT GAACCTTATC ACTTAAAGGA AGGA------ ----------
MK091090.1   CGGTAAATTA TTTAGACTGT ATTTATAGCA GTTT-CTGTG TTAAATATAG AAAGTTTTGT AAATCTTATC ACTTAGAGGA AGGAGAAGTC GTAACAAGGT
PQ326382.1   ---------- ---------- ---------- ---------- ---------- ---------- ---------- ---------- ---------- ----------
OP445729.1   ---------- ---------- ---------- ---------- ---------- ---------- ---------- ---------- ---------- ----------
JX469515.1   CGGTGAATTA TTCGGACCGC GATGT-TTCC GTA---AGGT TACGTCTAGG GAAGTTTTGT GAACCTTATC ACTTAAAGGA AGGAGAAGTC GTAACAAGGT
OP445726.1   ---------- ---------- ---------- ---------- ---------- ---------- ---------- ---------- ---------- ----------
AB520955.1   CGGTGAATTA TTCGGACCGT GATGT-TCCC GTT---AGGG AACGTCTAGG GAAGTTTTGT GAACCTTATC ACTTAAAGGA AGGAGAAGTC GTAACAAGGT
L24381.1 T   CGGTGAATTA TTCGGACCGT TTTGTGGCGC GTT---CGTG CCCGAAATGN GAAGTTTTGT GAACCTTAAC ACTTAGAGGA AGGAGAA--- ----------
U03070.1 T   CGGTGAATTA TTCGGACCGT TTTGTGGCGC GTT---CGTG CCCGAAATGG GAAGTTTTGT GAACCTTAAC ACTTAGAGGA AGGAGAAGTC GTAACAAGGT

             ....|....| ....|....| ....|....| .
                 1810       1820       1830
V4F          ---------- ---------- ---------- -
V4R          ---------- ---------- ---------- -
V9F          ---------- ---------- ---------- -
V9R          ----GTAGGT GAACCTGCRG AAGG------ -
KP666162.1   TTCCGTAGGT GAACCTGCAG AAGGATC--- -
AB478320.1   ---------- ---------- ---------- -
MK091090.1   TTCCGTAGGT GAACCTGCGG AAGGATCATT C
PQ326382.1   ---------- ---------- ---------- -
OP445729.1   ---------- ---------- ---------- -
JX469515.1   TTCCGTAGGT GAACCTGCAG AAGGATC--- -
OP445726.1   ---------- ---------- ---------- -
AB520955.1   TTCCGTAGGT GAACCTGCAG AAGGATC--- -
L24381.1 T   ---------- ---------- ---------- -
U03070.1 T   TTCCGTAGGT GAACCTGCAG AAGGATCA-- -
```

Table S3. Taxonomic classification of eukaryotes in ticks according to relative abundance of V4 and V9 reads.

Variables	V4 region		V9 region	
	Species	Relative abundance (%)	Species	Relative abundance (%)
Family Ixodidae	*Haemaphysalis longicornis*	37.990	*Haemaphysalis longicornis*	69.886
	Haemaphysalis nepalensis	27.705	*Ixodes scapularis*	29.971
	Ixodes pavlovskyi	24.177		
	Amblyomma hebraeum	9.900		
	Ixodes scapularis	0.028		
Subtotal		99.80		99.86
Non-Family Ixodidae	*Hepatozoon canis*	0.110	Other	0.055
	uncultured eukaryote	0.032	*Digitaria exilis*	0.025
	Other	0.017	*Ooctonus* sp.	0.019
	uncultured *Glomeromycotina*	0.011	*Hepatozoon canis*	0.013
	Fusarium sp.	0.009	*Felis catus*	0.007
	Chloroidium laureanum	0.009	*Saitozyma pseudoflava*	0.005
	Mediolabrus comicus	0.006	uncultured *gregarine*	0.004
	uncultured fungus	0.005	*Dicyrtomina* cf.	0.004
	Theileria luwenshuni	0.002	*Phaeotremella lactea*	0.004
			Exophiala lecanii-corni	0.003
			Entomortierella parvispora	0.002
			Basidiobolus sp.	0.002
Subtotal		0.20		0.14

Table S4. Taxonomic classification of eukaryotes in ticks based on the number of ASVs and abundance of V4 and V9 reads. By using bioinformatics, ASVs corresponding to Family Ixodidae were filtered out and only remaining ASVs are shown.

Species	ASV number (%)		Abundance (%)	
	V4	V9	V4	V9
Chloroidium laureanum	1 (8.33)	0 (0)	13 (4.56)	0 (0)
Fusarium sp.	1 (8.33)	0 (0)	13 (4.56)	0 (0)
Hepatozoon canis	1 (8.33)	1 (7.69)	156 (54.74)	19 (9.31)
Mediolabrus comicus	1 (8.33)	0 (0)	9 (3.16)	0 (0)
Theileria luwenshuni	1 (8.33)	0 (0)	3 (1.05)	0 (0)
Unclassified	2 (16.67)	2 (15.38)	24 (8.42)	78 (38.24)
uncultured *Glomeromycotina*	1 (8.33)	0 (0)	15 (5.26)	0 (0)
uncultured eukaryote	3 (25)	0 (0)	45 (15.79)	0 (0)
uncultured fungus	1 (8.33)	0 (0)	7 (2.46)	0 (0)
Basidiobolus sp.	0 (0)	1 (7.69)	0 (0)	3 (1.47)
Dicyrtomina cf.	0 (0)	1 (7.69)	0 (0)	6 (2.94)
Digitaria exilis	0 (0)	1 (7.69)	0 (0)	35 (17.16)
Entomortierella parvispora	0 (0)	1 (7.69)	0 (0)	3 (1.47)
Exophiala lecanii-corni	0 (0)	1 (7.69)	0 (0)	4 (1.96)
Felis catus	0 (0)	1 (7.69)	0 (0)	10 (4.9)
Ooctonus sp.	0 (0)	1 (7.69)	0 (0)	27 (13.24)
Phaeotremella lactea	0 (0)	1 (7.69)	0 (0)	6 (2.94)
Saitozyma pseudoflava	0 (0)	1 (7.69)	0 (0)	7 (3.43)
uncultured gregarine	0 (0)	1 (7.69)	0 (0)	6 (2.94)
Sum	12	13	285 (100)	204 (100)

Table S5. PCR positive samples for *T. gondii* identified by targeting B1 and 18S rRNA genes.

Tick pool ID	Target gene	
	B1	18S rRNA*
Tick-C5	+	+
Tick-C7	+	-
Tick-C9	+	+
Tick-C17	+	-
Tick-C21	+	+
Tick-C27	+	-
Tick-C30	+	-
Tick-C32	+	-
Tick-C40	+	-
Tick-C47	+	-
Tick-C65	+	-
Tick-C108	+	-
Tick-C229	+	-
Tick-C232	+	-
Tick-C272	+	-
Tick-J20	+	-
C22-81	+	-
J22-40	+	-
J22-169	+	-
No. of positive samples	19	3

*Nested PCR was done as described in "Detection of *Toxoplasma gondii* DNA in heart tissue from common marmoset (*Callithrix jacchus*) monitored for yellow fever and rabies in Pernambuco state, Northeastern of Brazil. *Vet Parasitol Reg Stud Reports*. 2020. doi: 10.1016/j.vprsr.2020.100447".
